# Caracterización de pacientes con hipotiroidismo congénito en el Hospital Universitario San Ignacio entre 2001 y 2017

**DOI:** 10.7705/biomedica.5334

**Published:** 2020-06-30

**Authors:** Lorena Peñaloza, Catalina Forero, Camila Céspedes

**Affiliations:** 1 Hospital Universitario San Ignacio, Pontificia Universidad Javeriana, Bogotá, D.C., Colombia Pontificia Universidad Javeriana Hospital Universitario San Ignacio Pontificia Universidad Javeriana BogotáD.C Colombia

**Keywords:** hipotiroidismo congénito/epidemiología, incidencia, tamización neonatal, tirotropina, tiroxina, recién nacido, Colombia, Congenital hypothyroidism/epidemiology, incidence, neonatal screening, thyrotropin, thyroxine, infant, newborn, Colombia

## Abstract

**Introducción.:**

El hipotiroidismo congénito es una causa prevenible de discapacidad cognitiva. Dada la ausencia de signos y síntomas al nacer, es necesario hacer pruebas de tamización para detectarlo. Su incidencia oscila entre uno de cada 2.500 y uno de cada 6.000 nacidos vivos.

**Objetivo.:**

Describir las características antropométricas y demográficas de los participantes, así como medir la concentración de tirotropina (TSH) en sangre de cordón umbilical y de TSH y tiroxina libre (T4 libre) en el suero de los recién nacidos positivos en la prueba de tamización y de aquellos con hipotiroidismo congénito confirmado.

**Materiales y métodos.:**

Se hizo un estudio observacional retrospectivo de un periodo de 17 años mediante la revisión de los registros de laboratorio clínico y las historias para establecer las características demográficas y antropométricas en el momento del nacimiento.

**Resultados.:**

Se analizaron 41.494 recién nacidos. Se encontraron 217 (0,52 %) recién nacidos con prueba positiva de tamización, 19 (8,76 %) de ellos con diagnóstico confirmado mediante pruebas séricas (TSH y T4 libre), para una incidencia de uno por cada 2.183 nacidos vivos. El 78,95 % de los casos de hipotiroidismo congénito correspondió a nacidos a término, el promedio de la edad gestacional fue de 37,3 semanas, similar al de quienes no lo presentaban. No hubo diferencia en el promedio de peso ni en la talla al nacer entre los afectados y los no afectados. La concentración de TSH en sangre de cordón umbilical fue significativamente mayor en los casos de hipotiroidismo congénito que en los recién nacidos sanos.

**Conclusiones.:**

La incidencia de hipotiroidismo congénito fue similar a la encontrada en los estudios consultados. No hubo diferencias clínicas relevantes entre los casos confirmados y los descartados, lo que resalta la pertinencia de la tamización neonatal para el diagnóstico temprano y el tratamiento oportuno.

## Abstract (en)

**Introduction:**

Congenital hypothyroidism is a preventable cause of cognitive disability. Due to the absence of symptoms and signs in the newborn, it is necessary to perform screening tests. Its incidence ranges between 1:2,500 and 1:6,000.

**Objective:**

To describe the anthropometric and demographic characteristics, as well as the cord TSH levels, serum TSH, and serum T4L levels of the positive patients during screening and patients with confirmed congenital hypothyroidism.

**Materials and methods:**

We conducted a retrospective observational study for 17 years based on the review of clinical laboratory and medical records to describe the demographic and anthropometric characteristics of the patients at the time of diagnosis.

**Results:**

We analyzed 41,494 newborns in the 17 years of follow-up; 217 (0.52%) were positive in the screening test and the diagnosis was confirmed by serum tests (TSH and free T4) in 19 cases (8.76%) for an incidence of one for every 2,183 live births; 78.95% of the children with congenital hypothyroidism were born full-term and the average gestational age was 37.3 weeks, similar to that of those with no congenital hypothyroidism. There was no difference in the average weight and height at birth between the children with the condition and those who did not have it. TSH in the cord in the cases of congenital hypothyroidism was significantly higher than in the discarded cases.

**Conclusions:**

The incidence of congenital hypothyroidism was similar to that found in the literature. There were no relevant clinical differences between confirmed and ruled out cases reflecting the relevance of neonatal screening.

El hipotiroidismo congénito es la principal causa de retardo mental prevenible en la población pediátrica ^1^. Sin embargo, dado que solo cerca del 5 % de los pacientes presenta sintomatología y que esta es inespecífica, su sospecha y diagnóstico clínico temprano son difíciles ^2^^-^^4^. Entre las principales consecuencias de esta condición se encuentran la discapacidad cognitiva y las alteraciones del crecimiento, lo que conlleva un incremento en los costos de salud individuales y colectivos a corto y largo plazo ^2^, razón por la cual se crearon programas de tamización a nivel mundial. El programa se viene implementando en Colombia en todos los recién nacidos desde el 2000 y es obligatorio para todas las entidades prestadoras de servicios de salud ^5^. La incidencia de esta enfermedad oscila entre 1:2.500 y 1:6.000 dependiendo de la población observada ^1^^,^^6^^,^^7^. El programa de tamización en el país ha sido supervisado por el Ministerio de Salud y Protección Social durante los últimos 18 años.

En este marco, el objetivo del presente estudio fue describir las características clínicas y demográficas de los pacientes con hipotiroidismo congénito detectados gracias al programa de tamización en el Hospital Universitario San Ignacio de Bogotá entre el 2001 y el 2017, así como determinar la frecuencia de los casos con base en los datos correspondientes a un periodo mayor que el de otros estudios previos a nivel nacional y regional. 

## Materiales y métodos

Se hizo un estudio observacional descriptivo y retrospectivo de la población de recién nacidos del Hospital Universitario San Ignacio de Bogotá. Se revisaron los registros manuales y los del sistema electrónico de historias clínicas de todas las mediciones de tirotropina (TSH) en sangre de cordón umbilical hechas como parte del programa obligatorio de tamización para hipotiroidismo congénito en los recién nacidos en esta institución, entre abril del 2001 y abril del 2017. El programa fue instaurado en el 2001 y, desde entonces, se lleva un registro de todas las pruebas practicadas, así como de los resultados obtenidos y del estado final de diagnóstico de hipotiroidismo, confirmado o descartado por las pruebas séricas para TSH y tiroxina libre (T4 libre).

Se detectaron los pacientes con resultado positivo en las pruebas de TSH en sangre de cordón umbilical y se recolectaron los datos de las siguientes variables: edad gestacional, peso al nacer, talla al nacer, sexo (masculino, femenino), valor de TSH en sangre de cordón umbilical, valores confirmatorios de TSH y de T4 libre en suero, confirmatorios y estado diagnóstico final (hipotiroidismo congénito confirmado o descartado). La clasificación antropométrica se hizo según la curva Intergrowth, así: peso adecuado para la edad gestacional (percentil entre 10 y 90), grande para la edad gestacional (percentil mayor de 90) o pequeño para la edad gestacional (percentil menor de 10) ^8^.

Se describieron las variables cuantitativas usando medidas de tendencia central y de dispersión (media y desviación estándar), en tanto que las variables cualitativas se describieron usando frecuencias y proporciones.

Se hizo un análisis exploratorio en el que se compararon las variables según los subgrupos de hipotiroidismo congénito confirmado, descartado y no confirmado. La comparación entre las variables continuas se hizo utilizando la prueba pareada t de Student o la prueba de Wilcoxon de suma de rangos dependiendo de la distribución de la variable y, para las variables categóricas, se utilizó una prueba de comparación de proporciones. Se usaron los programas Excel™ y Stata™ para el análisis de los datos.

### Consideraciones éticas

El presente trabajo fue aprobado por el Comité de Ética Institucional del Hospital Universitario San Ignacio.

## Resultados

Se revisaron 41.494 registros de pruebas de TSH en sangre de cordón umbilical tomadas como parte del programa obligatorio de tamización para hipotiroidismo congénito en recién nacidos en el Hospital Universitario San Ignacio de Bogotá entre abril del 2001 y abril del 2017.

Se encontraron 217 (0,52 %) recién nacidos con prueba de tamización positiva, pero solo en 19 (8,76 %) casos se obtuvo confirmación del diagnóstico de hipotiroidismo congénito con pruebas séricas (TSH y T4 libre), lo que representa una incidencia acumulada de 0,00046 durante los 17 años de seguimiento (aproximadamente, 4,6 por cada 10.000 o 1 por cada 2.183 nacidos vivos). En 152 (70,05 %) pacientes, se descartó el diagnóstico de hipotiroidismo congénito y en 21,2 % de los pacientes no se pudo verificar si se confirmó o se descartó el diagnóstico. A siete pacientes en este último grupo no se les hizo prueba de confirmación serológica (3,23 %) (cuadro 1). En el cuadro 2 se describen las características demográficas y los valores de TSH de este grupo.

**Cuadro 1:** Estado final del diagnóstico en la población con prueba positiva de tamización para hipotiroidismo congénito por TSH en sangre de cordón umbilical (n=217)

Diagnóstico final en pacientes con tamización positiva	n (%)
Hipotiroidismo congénito confirmado	19 (8,76)
Hipotiroidismo congénito descartado	152 (70,05)
Diagnóstico desconocido	46 (21,1)

DE: desviación estándar

**Cuadro 2:** Características de la población con prueba positiva de tamización para hipotiroidismo congénito por TSH en sangre de cordón umbilical

	n (%)
Edad gestacional en semanas, promedio (DE)	38 (±1,79)
Sexo femenino, n (%)	100 (46)
Peso, promedio (DE)	2.910 (±484)
TSH en sangre de cordón umbilical en µUl/ml, promedio (DE)	37,7 (±47)

DE: desviación estándar

En cuanto a las características de los casos confirmados en el momento del nacimiento, se encontró que el 78,95 % nació a término, con un promedio de edad gestacional de 37,31 ± 2,6 semanas, y el 21,05 % nació antes de término, entre las semanas 30 y 36 de gestación. Estos porcentajes fueron similares en los pacientes en quienes se descartó el hipotiroidismo congénito.

En los 217 pacientes con resultado positivo en la prueba de tamización, se encontró un peso adecuado al nacer, con un promedio de 2.910 g (desviación estándar, DE: ±428 g), en tanto que en el 15,21 % hubo bajo peso al nacer. En los casos en que se confirmó el hipotiroidismo congénito, el promedio de peso fue de 2.732 g (DE: ±628 g), y el 21,05 % (n=4) tuvo un peso menor de 2.500 g, porcentaje correspondiente a cuatro pacientes prematuros. Solo un recién nacido de este último grupo fue pequeño para la edad gestacional.

El promedio de la talla al nacer de los recién nacidos con hipotiroidismo congénito fue de 48 cm (DE: ±2,33), con un rango intercuartílico de 47 a 49,4 cm entre los percentiles 25 y 75. En cinco de los casos no se encontró el registro de la talla.

En cuanto al estado del diagnóstico final, la media de TSH en sangre de cordón umbilical de la población con hipotiroidismo congénito confirmado fue significativamente mayor en comparación con la de aquellos con hipotiroidismo descartado, así como la de TSH sérica.

La media de TSH en sangre de cordón umbilical para el grupo de pacientes con hipotiroidismo congènito descartado fue de 26,5 μUl/ml (DE: ±9,82), en tanto que, en los casos confirmados, fue de 142 μUl/ml (DE: ±112,2) (cuadro 3). Resultados similares se encontraron al comparar la TSH sérica en ambos grupos: 3,3 μUl/ml (DE: ±2,54) *Vs*. 174,1 μUl/ml (DE: ±161,5), respectivamente. En cuanto a los niveles de T4 libre, también hubo diferencias significativas: 0,71 ng/dl (DE: ±0,49) en los casos confirmados *Vs*. 1,74 ng/dl (DE: ±1,63) en el grupo de los descartados.

**Cuadro 3:** Comparación de variables en pacientes con prueba positiva de tamización e hipotiroidismo congénito confirmado y descartado

	Hipotiroidismo congénito confirmado	Hipotiroidismo congénito descartado
	**(n=19)**	(n=152)
	n (%)	n (%)
Edad gestacional en semanas, promedio (DE)	37,3 (±2,6)	38,15 (±1,4)
Nacimiento a término, n (%)	15 (78,95)	138 (90,79)
Nacimiento prematuro, n (%)	4 (21,05)	14 (9,21)
Peso, promedio (DE)	2,732 (628)	2969,6 (±428)
Bajo peso al nacer, n (%)	4 (21,05)	18 (11,84)
Sexo femenino, n (%)	8 (42,1)	71 (46,7)
TSH en sangre de cordón umbilical en µUl/ml, promedio (DE)	142,1 (±112,3)	26,44 (±9,82)
TSH sérica en µUl/ml, promedio (DE)	174,1 (±161,6)	3,37 (±2,54)
T4 libre sérica en ng/dl, promedio (DE)	0,71 (±0,49)	1,74 (±1,63)

DE: desviación estándar

Se detectaron cuatro pacientes en quienes el valor de T4 libre sérica se encontraba cercano al valor de referencia normal; en tres de ellos se sospechó ectopia tiroidea dado los altos niveles de TSH sérica que presentaban (entre 67 y 262 μUl/ml) y en uno de ellos se confirmó dicha sospecha. En un paciente, la TSH sérica estaba moderadamente elevada (27,19 μUl/ml), pero teniendo en cuenta las implicaciones neurológicas del diagnóstico se optó por darle tratamiento. Estos pacientes no continuaron el seguimiento en el Hospital Universitario San Ignacio de Bogotá.

La proporción de hombres fue mayor, con una relación entre hombres y mujeres de 1,4:1 en el grupo de casos positivos.

## Discusión

En este estudio se utilizaron los datos recolectados en los registros del programa de hipotiroidismo congénito entre abril del 2001 y abril del 2017. Se encontró una incidencia de 1 por cada 2.183 nacidos vivos, la cual está en el rango de las diferentes incidencias reportadas a nivel mundial, nacional y regional. Se encontró, asimismo, que la TSH en sangre de cordón umbilical como método de tamización es muy sensible, aunque poco específica, y cumple con su cometido.

A nivel mundial se han reportado incidencias entre 1:3.000 y 1:4.000 ^2^, que varían según la población estudiada y la época de tamización. En un estudio realizado en el Hospital Materno-Infantil de Bogotá, se reportó una frecuencia de un caso por cada 3.448 nacidos vivos ^4^. En otro estudio realizado en varias instituciones prestadoras de servicios de salud en la misma ciudad entre el 2001 y el 2009, se reportó una incidencia de 1 por cada 3.801 nacidos vivos ^9^, en tanto que, en otros países de Latinoamérica, como Argentina, la incidencia fue de 1 por cada 3.108 y 2.367 nacidos vivos (Acosta J, Prieto J. Análisis retrospectivo de 10 años del programa de tamizaje neonatal para hipotiroidismo congénito de la red distrital de salud de Bogotá. Iatreia. 2010. S-58. Memorias, XI Congreso Colombiano de Genética Humana, Medellín, 6 a 8 de octubre de 2010).

Al comparar la población con prueba positiva de TSH en sangre de cordón umbilical y de TSH sérica negativa para hipotiroidismo congénito y aquella con diagnóstico confirmado de hipotiroidismo congénito, se encontró que la TSH en sangre de cordón umbilical fue acentuadamente más elevada en aquellos con hipotiroidismo congénito comparados con quienes no lo presentaban.

El punto de corte de TSH en sangre de cordón umbilical a partir del cual debe practicarse una prueba sérica confirmatoria es de 15 μUI/ml.

Según los resultados de este estudio, y de otros reportados en la literatura especializada, cuanto mayor sea el valor de TSH en sangre de cordón umbilical, más probable es el diagnóstico de hipotiroidismo congénito de origen primario. Según nuestros datos, se puede establecer que los valores de TSH en sangre de cordón umbilical por encima de 30 μUI/ml serían muy sugestivos de hipotiroidismo congénito primario.

Pese a ello, es importante señalar que el rango mínimo de TSH de tamización en el grupo de pacientes en los que se confirmó el diagnóstico de hipotiroidismo congénito primario, se situó en 23 μUI/ml, lo que respalda la pertinencia del punto de corte establecido. Asimismo, los valores de T4 libre sérica fueron mucho menores en pacientes con hipotiroidismo congénito, salvo en cuatro de ellos que presentaban valores de TSH sérica altos. Solo en uno de estos cuatro pacientes se pudo confirmar la sospecha de ectopia tiroidea, pero dado que el seguimiento de los tres pacientes no se hizo en el hospital, no se pudo establecer si alguno presentaba hipotiroidismo congénito transitorio o si realmente se trataba de una disgenesia tiroidea.

En cuanto al sexo, la proporción de hombres afectados fue ligeramente mayor, con una relación de 1,4:1, a diferencia de otras series en las que se describe una mayor frecuencia en el sexo femenino (1:2) ^9^^,^^10^, como la del estudio realizado entre 1995 y 1999 en Bogotá, en el que la proporción de niñas y niños fue de 9:1 ^4^.

Por otro lado, el promedio del peso al nacer fue menor en los pacientes con hipotiroidismo congénito, pero en ninguno de los grupos correspondió a bajo peso al nacer.

Entre las limitaciones del estudio, deben mencionarse las dificultades para recolectar todos los datos, dado que los registros de los primeros cinco años solo se encontraban en una base de datos manual y no fue posible cotejar la información con la historia clínica electrónica, por lo cual, en algunos casos, no se pudo verificar si el diagnóstico de hipotiroidismo congénito se había confirmado o descartado.

Entre las fortalezas están el tiempo del seguimiento del programa, 17 años, pues en el país no ha habido estudios publicados con tanto tiempo de seguimiento. La incidencia fue similar a la reportada a nivel mundial y estuvo en el rango reportado en diferentes poblaciones, es decir que esta incidencia no ha cambiado a lo largo del tiempo.

Las dificultades para obtener los datos evidencian la necesidad de sistematizar su registro, con el fin de facilitar el acceso a la información necesaria para el seguimiento de los pacientes y para futuros estudios de evaluación del impacto a mediano y largo plazo del programa de tamización. Asimismo, se abre un espacio para valorar, con las directivas del Hospital San Ignacio, la posibilidad de garantizar el seguimiento de los casos positivos.

Los resultados de este informe hacen parte de la primera fase de un estudio mayor. En una segunda fase, se pretende evaluar los resultados clínicos a largo plazo de los casos positivos de hipotiroidismo congénito.

Los resultados del presente estudio indican que la incidencia de hipotiroidismo congénito es similar a la encontrada en otros estudios a nivel nacional y en poblaciones de otras latitudes, y respalda la necesidad de hacer una prueba confirmatoria si la tamización es positiva. No hubo diferencias clínicas relevantes entre los casos y los pacientes sin hipotiroidismo congénito, lo que resalta la importancia de la tamización neonatal universal. El diagnóstico y el tratamiento tempranos son fundamentales para prevenir las secuelas neurológicas de esta condición.
